# Acute ethanol exposure inhibits silencing of cerebellar Golgi cell firing induced by granule cell axon input

**DOI:** 10.3389/fnint.2014.00010

**Published:** 2014-02-06

**Authors:** Paolo Botta, Aya Zucca, C. Fernando Valenzuela

**Affiliations:** Department of Neurosciences, University of New Mexico Health Sciences CenterAlbuquerque, NM, USA

**Keywords:** cerebellum, Golgi cell, interneuron, ethanol, metabotropic, glutamate, feedback, GABA

## Abstract

Golgi cells (GoCs) are specialized interneurons that provide inhibitory input to granule cells in the cerebellar cortex. GoCs are pacemaker neurons that spontaneously fire action potentials, triggering spontaneous inhibitory postsynaptic currents in granule cells and also contributing to the generation tonic GABA_A_ receptor-mediated currents in granule cells. In turn, granule cell axons provide feedback glutamatergic input to GoCs. It has been shown that high frequency stimulation of granule cell axons induces a transient pause in GoC firing in a type 2-metabotropic glutamate receptor (mGluR2)-dependent manner. Here, we investigated the effect ethanol on the pause of GoC firing induced by high frequency stimulation of granule cell axons. GoC electrophysiological recordings were performed in parasagittal cerebellar vermis slices from postnatal day 23 to 26 rats. Loose-patch cell-attached recordings revealed that ethanol (40 mM) reversibly decreases the pause duration. An antagonist of mGluR2 reduced the pause duration but did not affect the effect of ethanol. Whole-cell voltage-clamp recordings showed that currents evoked by an mGluR2 agonist were not significantly affected by ethanol. Perforated-patch experiments in which hyperpolarizing and depolarizing currents were injected into GoCs demonstrated that there is an inverse relationship between spontaneous firing and pause duration. Slight inhibition of the Na^+^/K^+^ pump mimicked the effect of ethanol on pause duration. In conclusion, ethanol reduces the granule cell axon-mediated feedback mechanism by reducing the input responsiveness of GoCs. This would result in a transient increase of GABA_A_ receptor-mediated inhibition of granule cells, limiting information flow at the input stage of the cerebellar cortex.

## Introduction

**Figure 1:**
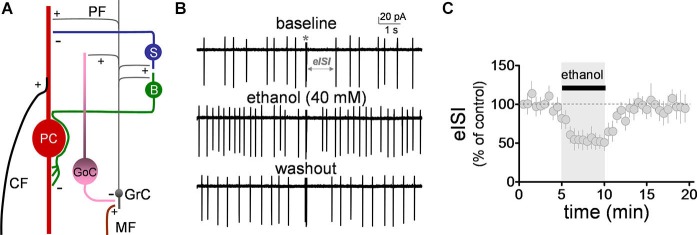
**Ethanol reversibly decreased the firing pause in Golgi cells. (A)** Schematic representation of the main components of the cerebellar cortex. A mossy fiber (MF) makes a synaptic connection with a granule cell (GrC) dendrite. The granule cell axon (both via the ascending and the parallel fiber (PF) segments), in turn, connects with a Golgi cell (GoC) apical dendrite, providing feedback excitation to the GoC, and driving feedback inhibition of the GrC. Under conditions of burst stimulation, synaptic transmission at GrC axon-to-GoC dendrite causes a pause in spontaneous action potential firing of GoCs, leading to transient disinhibition of GrCs. Not depicted are the basal dendrites of the GoC or the MF inputs to these dendrites. Also shown are the stellate (S) and basket (B) cells, which are excited by GrC axons and provide inhibitory input to the Purkinje cell (PC). The two glutamatergic inputs to the PC (i.e., the climbing fiber, CF, and PF) are also depicted. +, glutamatergic synapse; −, GABAergic synapse. **(B)** Sample traces corresponding to a loose-patch cell-attached recording from a Golgi cell. The baseline trace illustrates the pause in spontaneous firing of the Golgi cell induced by granule cell axon stimulation with a train of five stimuli (50 µs duration each) at 100 Hz (marked with an asterisk). The pause in firing is denoted as the evoked interspike interval (eISI). The middle trace shows that ethanol increased basal firing of the Golgi cell and reduced the duration of the pause. The bottom trace shows that this effect was reversible upon washout. **(C)** Pooled data from five Golgi cells illustrating the time course of the effect of ethanol on the eISI. Values were normalized with respect to the first 30 s of recording.

The cerebellum controls motor coordination, balance, muscle tone, motor learning, and cognition. These functions are mediated, in part, by neurons located in the cerebellar cortex, which receives excitatory input from the somatosensory system and the cerebral cortex (Ito, 2006). These excitatory inputs are relayed by glutamatergic mossy fibers originating in the brain stem and spinal cord. A mossy fiber makes synaptic connections with hundreds of cerebellar granule cells and thousands of these cells provide excitatory input to Purkinje neurons via the ascending portion of their axons as well as the parallel fibers (Figure 1A). The activity of cerebellar granule cells is regulated by inhibitory input provided by a specialized interneuron, the Golgi cell (GoC; Figure 1A). The typical GoC has a large soma (~20 µm) with 2–3 long apical dendrites that project into the molecular layer and several short basal dendrites located in the granule cell layer. The GoC axon is extensively ramified, making synaptic contacts with thousands of granule cell and unipolar brush cell dendrites (Ito, 2006; D’Angelo, 2008; Galliano et al., 2010). Studies with mice indicate that a significant portion of GoCs can co-express both GABAergic and glycinergic markers (Simat et al., 2007). However, the inhibitory postsynaptic currents at GoC-granule cell synapses are solely mediated by GABA_A_ receptors, whereas those at unipolar brush cells are mediated by both GABA_A_ and glycine receptors (Geurts et al., 2003).

GoCs provide both tonic and phasic GABA_A_ receptor-mediated inhibition to cerebellar granule cells. Tonic inhibition is mediated by extrasynaptic GABA_A_ receptors containing α_6_βδ subunits, activated by ambient GABA levels, generated, in part, by accumulation of GABA released from GoCs and perhaps also from lamellar astrocytes under certain experimental conditions (Lee et al., 2010; Diaz et al., 2011, Diaz et al., 2013). Phasic inhibition is primarily mediated by synaptic GABA_A_ receptors containing *α*_1_*βγ*2 subunits, activated by evoked or spontaneous action potential-dependent GABA release (Rossi et al., 2003). Evoked GABA release is triggered by mossy fiber or granule cell axon (i.e., both ascending and parallel fiber segments) stimulation of GoCs, whereas spontaneous GABA release is driven by spontaneous action potential firing of GoCs. GoCs display ongoing action potential firing activity at frequencies between 1–10 Hz in cerebellar slices (Dieudonne, 1998; Carta et al., 2004; Forti et al., 2006; Solinas et al., 2007; Botta et al., 2010), 2–30 Hz in anesthetized rats (Schulman and Bloom, 1981; Vos et al., 1999; Holtzman et al., 2006), ~8 Hz in awake rats (Huang et al., 2012) and 10–80 Hz in awake monkeys (Miles et al., 1980). Recent evidence suggests that GoCs form an extensive neuronal network, interconnected via gap junctions, that synchronously oscillates in the 5–30 Hz range, providing rhythmic inhibition to granule cells and ultimately controlling responsiveness to mossy fiber input (Dugue et al., 2009; Ros et al., 2009; Simões de Souza and De Schutter, 2011).

As mentioned above, granule cell axons provide excitatory input to GoCs and this, in turn, provides feedback inhibition to granule cells, decreasing responsiveness to mossy fiber excitatory input (Ito, 2006; Figure 1A). Importantly, studies suggest that this feedback loop may be transiently inhibited by glutamatergic inputs evoked by some sensory stimuli. Specifically, glutamate release evoked by high frequency stimulation of parallel fibers transiently suppresses GoC firing via activation of type-2 metabotropic glutamate receptors (mGluR2; Watanabe and Nakanishi, 2003). These receptors activate G protein-coupled inwardly-rectifying potassium (GIRK) channels, which hyperpolarize the membrane potential of GoCs (Watanabe and Nakanishi, 2003). It is thought that this transient suppression of GoC firing allows some mossy fiber inputs to readily excite granule cells without an opposing influence of phasic GABA_A_ receptor-mediated transmission from GoCs. *In vivo* studies have shown that GoCs typically respond to brief tactile stimuli by decreasing their spontaneous action potential firing rate, suggesting that this mechanism is physiologically important (Holtzman et al., 2006; Xu and Edgley, 2008).

Studies have shown that acute ethanol exposure increases spontaneous action potential firing of GoCs, resulting in an increase in both phasic and tonic GABAergic input to granule cells (Carta et al., 2004; Hanchar et al., 2005; Botta et al., 2010, Botta et al., 2012; Huang et al., 2012; Diaz et al., 2013). In this study, we investigated if acute ethanol exposure also modulates the transient suppression of GoC firing induced by granule cell axon stimulation.

## Materials and methods

For all the experiments, we used ethanol (95%, spectrophotometric grade) from Sigma Chemical Co. (St. Louis, MO). (2S)-2-Amino-2-[(1S,2S)-2-carboxycycloprop-1-yl]-3-(xanth-9-yl) propanoic acid (LY341495), 6-Imino-3-(4-methoxyphenyl)-1(6H)-pyridazinebutanoic acid hydrobromide (gabazine), [S-(R*,R*)]-[3-[[1-(3,4-Dichlorophenyl)ethyl]amino]-2-hydroxypropyl](cyclohexylmethyl) phosphinic acid (CGP 54626), 2S,2′R,3′R)-2-(2′,3′-Dicarboxycyclopropyl)glycine (DCG-IV), and DL-2-Amino-5-phosphonopentanoic acid were from Tocris-Cookson (Ellisville, MO). Tetrodotoxin was from EMD Millipore (Billerica, MA). All other chemicals were from Sigma.

### Brain slice preparation

All animal procedures were approved by the UNM-Health Sciences Center Institutional Animal Care and Use Committee and conformed to National Institutes of Health Guidelines. Experiments were performed in parasagittal vermis cerebellar slices that were prepared from postnatal day (P) 23-P26 male Sprague-Dawley rats (Harlan, Indianapolis, IN). Animals were euthanized by rapid decapitation under deep anesthesia with ketamine (250 mg/kg I.P.) and 200 µm thick slices were prepared with a vibrating slicer (Technical Products International, St. Louis, MO). Slices were cut in cold solution containing (in mM): 220 sucrose, 26 NaHCO_3_, 10 glucose, 6 MgSO_4_, 2 KCl, 1.25 NaH_2_PO_4_, 0.2 CaCl_2_ and 0.43 ketamine; this solution was pre-equilibrated with 95% O_2_ plus 5% CO_2_. Immediately after this procedure, slices were transferred to a chamber containing artificial cerebrospinal fluid (ACSF) and allowed to recover at 35–36°C for 35 min, followed by storage at room temperature for at least 1.5 h before the start of recordings. ACSF contained (in mM): 126 NaCl, 2 KCl, 1.25 NaH_2_PO_4_, 1 MgSO_4_, 26 NaHCO_3_, 2 CaCl_2_, and 10 glucose equilibrated with 95% O_2_ plus 5% CO_2_. Slices were transferred to a recording chamber perfused with ACSF at a rate of 2–3 ml/min and maintained at 32–33°C.

### Loose-patch cell-attached electrophysiological recordings

Neurons were visualized using infrared-differential interference contrast microscopy and recordings were performed with a Multiclamp 700B amplifier (Molecular Devices, Sunnyvale, CA). GoCs were primarily identified on the basis of their location in the granule cell layer, larger size when compared to granule cells, and the presence of spontaneous action potential firing. Patch pipettes had resistances of 3–5 MΩ. Each slice was exposed once to ethanol and the duration of ethanol exposure was limited to 5 min to avoid the development of rapid tolerance. The loose-patch cell-attached configuration (seal resistance = 8–30 MΩ) was used to record action currents and the firing pause. The patch pipettes were filled with regular ACSF and the holding potential was 0 mV; the holding potential in loose-patch cell-attached experiments does not significantly affect the GoC resting membrane potential because most of the current generated by the amplifier leaks across the loose seal (Perkins, 2006). The pause in GoC firing was induced with a train of five stimuli (50 µs duration each) at 100 Hz (train duration approximately 40 ms) delivered every 30 s. Stimulation was achieved with a concentric bipolar stimulating electrode placed in the inner half of the molecular layer. The pause length was calculated as the time between the stimulation train and the longest interspike interval (ISI) that was observed after the train (i.e., spikes that occurred soon after the high frequency train were not considered; see Figure 3A below for an example).

### Whole-cell patch-clamp recordings

The whole-cell patch-clamp configuration was used to record GIRK channel-mediated currents evoked by the mGluR2 agonist, DCG-IV. The recordings were performed as described in the pre-vious section with the exception that pipettes were filled with an internal solution containing (in mM): 90 KH_2_PO_4_, 10 KCl, 10 2-[4-(2-hydroxyethyl)piperazin-1-yl]ethanesulfonic acid (HEPES), 10 1,2-bis(o-aminophenoxy)ethane-N,N,N′,N′-tetraacetic acid (BAPTA), 4 MgCl_2_, 0.4 GTP, 2 ATP, and 5 phospho-creatine (pH 7.4 with KOH) (Watanabe and Nakanishi, 2003). The mem-brane potential was held at −70 mV. The ACSF contained antag-onists of GABA_A_ receptors (gabazine, 25 µM), GABA_B_ receptors ([S-(R*,R*)]-[3-[[1-(3,4-Dichlorophenyl)ethyl]amino]-2-hydroxypropyl] (cyclohexylmethyl) phosphinic acid; CGP 54626, 10 µM), glycine receptors (strychinine, 1 µM), Na^+^ channels (tetrodotoxin, 1 µM), ionotropic glutamate receptors (kynurenic acid, 1 mM), and NMDA receptors (DL-2-Amino-5-phosphonopentanoic acid, 50 µM). DCG-IV (10 µM) was applied in the bath for 30 s. After a steady-state current was observed, the mGluR2 antagonist, LY341495 (0.5 µM), was bath-applied for 2–3 min to block the response. Currents were evoked every 10–12 min.

### Perforated-patch electrophysiological recordings

The perforated-patch configuration was used to further study the effect of ethanol on the firing pause and to characterize the effect of current injection on firing frequency and pause. An amphotericin-B stock solution was made fresh daily (2 mg/ml in dimethylsulfoxide). The stock solution was sonicated for ~15 min, then continuously vortexed at a low speed for the duration of the recording session. The microelectrode tips were prefilled with internal solution containing (in mM): 135 K-gluconate, 5 KCl, 10 HEPES, 0.2 Ethylene glycol-bis(2-aminoethylether)-N,N,N′,N′-tetraacetic acid (EGTA), 4.6 MgCl_2_, 0.1 CaCl_2_, 4 Na_2_-ATP and 0.4 Na-GTP (pH 7.35 adjusted with KOH) and then backfilled with the same internal solution containing 10 µg/ml of amphotericin-B. The access resistance was used to monitor the progression of perforation, which was considered complete when it was between 40–80 MΩ.

Recordings were performed in the current-clamp mode and currents were injected as indicated. The firing pause was induced as described in the previous section.

### Data analyses

Data were filtered at 2 kHz and acquired at 5–50 kHz with a Digidata 1322A digitizer and pClamp-9 (Molecular Devices, Sunnyvale, CA). Data were analyzed with Clampfit-9 (Molecular Devices) and MiniAnalysis-6.0.3. (Synaptosoft, Decatur, GA). Data were statistically analyzed with Prizm version 4 or 5 (GraphPad, San Diego, CA) and are presented as mean ± SEM. To test for normal distribution, data were initially analyzed with the Pearson omnibus normality test. If data followed a normal distribution, these were analyzed using parametric tests. If this was not the case, then non-parametric tests were used. The unit of determination was defined as a slice.

## Results

### Ethanol reversibly decreases the duration of the firing pause

We initially evaluated the effect of ethanol on the firing pause using the loose-patch cell-attached configuration. The pause in spontaneous GoC firing (denoted as the evoked inter-spike interval, eISI) was elicited by a train of high frequency stimulation as described in Section Loose-patch Cell-attached Electrophysiological Recordings. The pause length was calculated as described in Section Loose-patch Cell-attached Electrophysiological Recordings. The high frequency stimulation train elicited an eISI of 2.4 ± 0.6 s (*n* = 5; see Figure 1B for sample trace). Application of ethanol (40 mM) significantly decreased the eISI (Figures 1B–C; average of 5 min baseline eISI = 99 ± 8.4%; average of 5 min ethanol eISI = 53.4 ± 11.4%; paired student *t*-test, *t* = 6.25, *df* = 4, *p* = 0.003; *n* = 5). In agreement with previous reports (Carta et al., 2004; Botta et al., 2010, Botta et al., 2012), ethanol also increased the GoC firing frequency by 1.2 ± 0.2 Hz (one sample *t*-test vs. 0, *t* = 6.6, *df* = 4, *p* = 0.002; *n* = 5).

**Figure 2:**
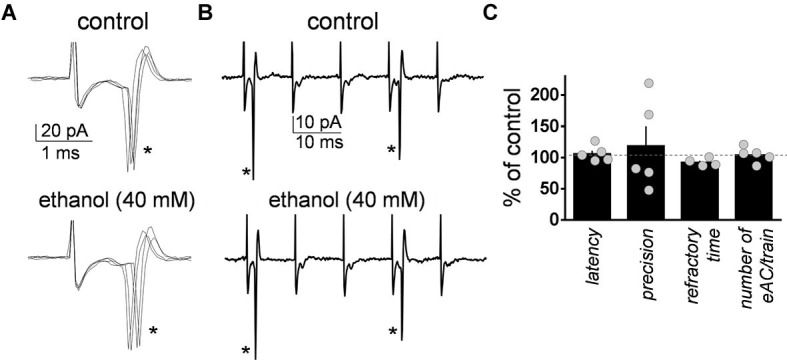
**Ethanol did not significantly affect the characteristics of the action currents evoked in Golgi cells by high frequency stimulation of granule cell axons. (A)** Sample traces corresponding to a loose-patch cell-attached recording from a Golgi cell illustrating the action currents evoked by the first stimulus (marked with an asterisk) of the five stimuli train (50 µs duration each at 100 Hz) in the absence and presence of 40 mM ethanol. **(B)** Same as in **A** but at a more compressed time scale. Note the two action currents (indicated by the asterisks) triggered by the five stimuli train. **(C)** Summary graph illustrating the lack of effect of ethanol on latency, precision, refractory time, and number of evoked action currents (eAC) per train (*n* = 4–5).

As a control, we assessed the effect of ethanol on the changes in GoC excitability elicited by the train of high frequency stimulation. The latency was calculated as the time between the stimulus artifact and the sodium current peak (Figure 2A). The precision calculated as the standard deviation of the latency (Figure 2A). The refractory time was calculated as the time between the first two action currents evoked by the train of high frequency stimulation (Figure 2B). The average number of action currents triggered by the train of 5 stimuli was 2.3 ± 0.6 (*n* = 5; Figure 2B). Figure 2C shows that ethanol did not change the latency (one sample *t*-test vs. 100, *t* = 0.95, *df* = 4, *p* = 0.39; *n* = 5), precision (one sample *t*-test vs. 100, *t* = 0.55, *df* = 4, *p* = 0.6; *n* = 5), refractory time (one sample *t*-test vs. 100, *t* = 2.57, *df* = 3, *p* = 0.08; *n* = 4), or number of evoked action currents elicited by the train of high frequency stimulation (one sample *t*-test vs. 100, *t* = 0.65, *df* = 4, *p* = 0.54; *n* = 5).

### Ethanol reduces the firing pause independently of mGluR2 receptors

**Figure 3:**
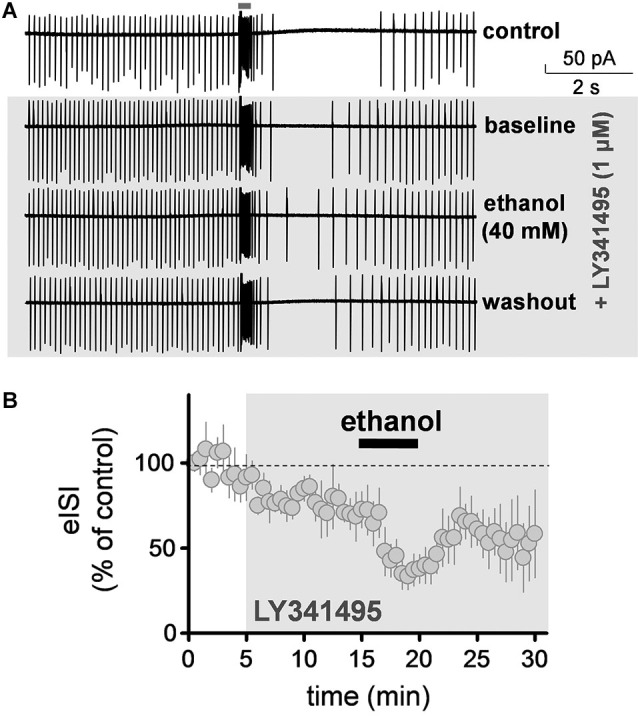
**Ethanol decreased the firing pause duration in presence of an mGluR2 antagonist. (A)** Sample traces corresponding to a loose-patch cell-attached recording from a Golgi cell illustrating that the mGluR2 blocker, LY341495 (1 µM) reduced the duration of the pause evoked by stimulation of granule cell axons with a train of five stimuli (50 µs duration each at 100 Hz). Ethanol (40 mM) caused a further reduction in the pause duration in presence of this blocker and this was reversible upon washout. **(B)** Summary of results obtained with nine Golgi cells illustrating the time course of the changes in the pause (i.e., evoked interspike interval; eISI) induced by LY341495 and ethanol. Values were normalized with respect to the first 30 s of recording.

We evaluated the effect of ethanol on the firing pause in presence of a saturating concentration of the mGluR2 antagonist, LY341495 (1 µM). These experiments were also performed in the loose-patch cell-attached configuration. LY341495 did not significantly affect baseline firing of GoCs (109.5 ± 8.4% of control; one sample *t*-test vs. 100, *t* = 1.128, *df* = 8, *p* = 0.29; *n* = 9; see Figure 3A for sample traces). However, it significantly decreased the duration of the firing pause (Figure 3B; average of 5 min baseline eISI = 99.7 ± 9%; average of 10 min LY341495 eISI = 76.6 ± 6.2%; paired student *t*-test, *t* = 7.73, *df* = 4, *p* = 0.0015; *n* = 5). In presence of LY341495, ethanol (40 mM) decreased the firing pause to a similar extent as in the absence of this agent (Figure 3B; average of 10 min LY341495 eISI = 76.6 ± 6.2%; average of 5 min ethanol eISI = 38.7 ± 8.1 %; paired student *t*-test, *t* = 13.95, *df* = 4, *p* = 0.0002; the eISI in presence of ethanol was 48.2 ± 6.8% of the eISI in presence of LY34195; *n* = 5; compare with Figure 1B).

**Figure 4:**
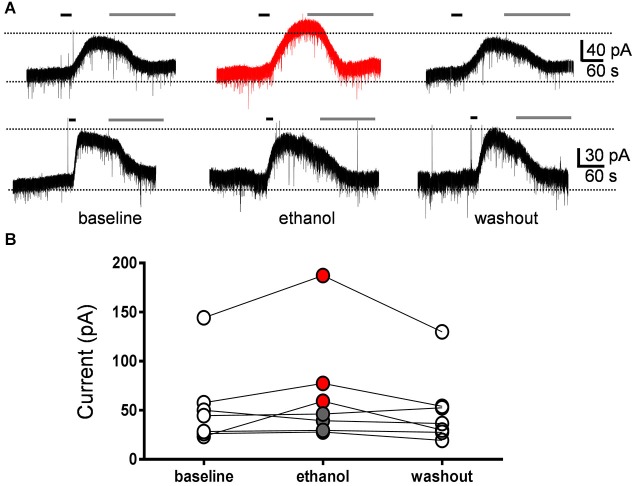
**Currents evoked by the mGluR2 agonist, DCG-IV, were not significantly affected by ethanol. (A)** Sample traces corresponding to a whole-cell voltage-clamp recording from a Golgi cell illustrating the outward currents evoked by a 30 s bath application of DCG-IV (10 µM; black line). Subsequent application of LY341495 (0.5 µM; gray line) blocked the DCG-IV-evoked currents. Currents were evoked at 10–12 min intervals. The top trace corresponds to a GoC recording where ethanol reversibly potentiated the DCG-IV-evoked current and the lower trace to a recording where ethanol had no effect. **(B)** Graph summarizing the results obtained in seven Golgi cell recordings, illustrating that ethanol potentiated DCG-IV-evoked currents in three cells (red circles) and did not change them in four cells (gray circles). On average, the DCG-IV-evoked currents in presence of ethanol were 130 ± 21% with respect to the average of baseline and washout responses.

We directly assessed the effect of ethanol on GIRK currents evoked by mGluR2 stimulation. These currents were evoked by a 30 s bath application of the selective mGluR2 agonist, DCG-IV (10 µM), followed by application of the mGluR2 antagonist, LY341495 (0.5 µM) to block the responses (Figure 4A). The effect of acute exposure to 40 mM ethanol on these currents was tested in seven GoCs. Ethanol reversibly increased the DCG-IV-evoked current in three GoCs and had no effect in the other four GoCs (Figures 4A, B). On average, the DCG-IV-evoked current was 130 ± 21% of the average of control and washout responses (Wilcoxon signed rank test vs. theoretical median of 100; sum of signed ranks (W) = 20, sum of positive ranks = 24, sum of negative ranks = −4; *p* = 0.10; *n* = 7).

### Ethanol reduces the firing pause by depolarizing the membrane potential and increasing action potential firing of Golgi cells (GoCs)

**Figure 5:**
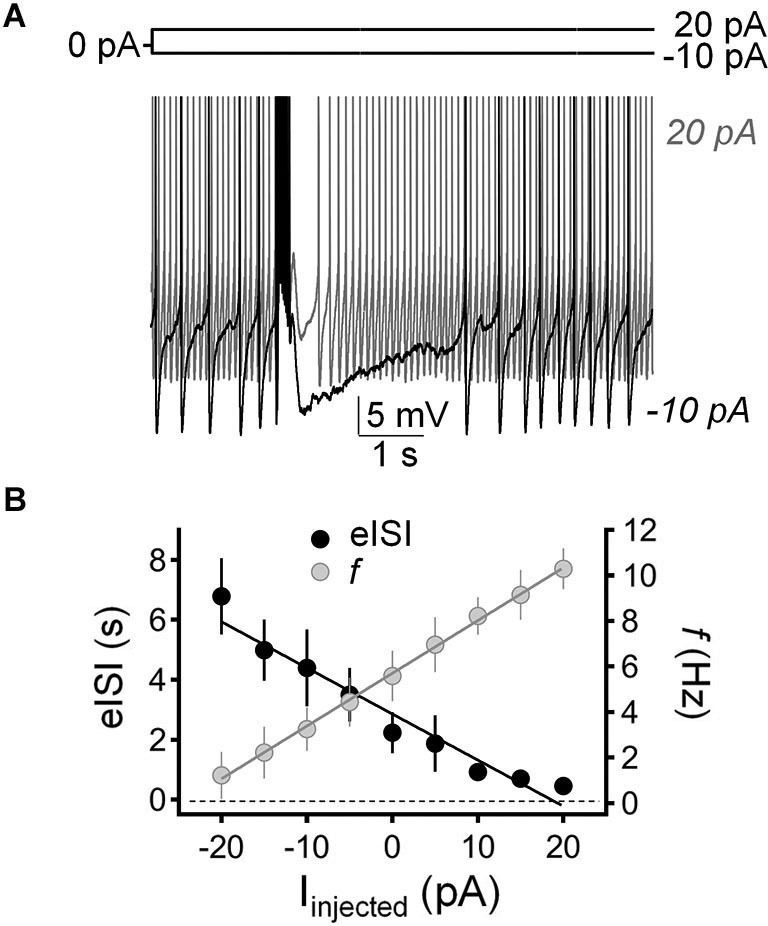
**Injection of hyperpolarizing or depolarizing current induced reciprocal changes in Golgi cell firing frequency and pause duration. (A)** Sample traces corresponding to perforated-patch current-clamp recordings from a Golgi cell illustrating the effect of hyperpolarizing current injection (−10 pA; black trace) and depolarizing current injection (20 pA, gray trace) on spontaneous firing frequency and pause duration. The pause was induced with a train of five stimuli (50 µs duration each at 100 Hz). **(B)** Summary of results obtained in recordings from 9 Golgi cells illustrating the inverse relationship between changes in firing frequency (*f*, gray circles) and pause duration (evoked interspike interval; eISI, black circles) in response to current injection (І_injected_).

We tested the impact of current injection on the firing pause using the perforated-patch current-clamp configuration. Figure 5A shows that injection of depolarizing current (20 pA) both increased the action potential firing frequency and reduced the duration of the pause. Conversely, injection of hyperpolarizing current (−10 pA) both reduced GoC firing and increased the duration of the pause. Figure 5B shows that there is an inverse relationship between the impact of current injection on firing frequency and pause duration.

**Figure 6:**
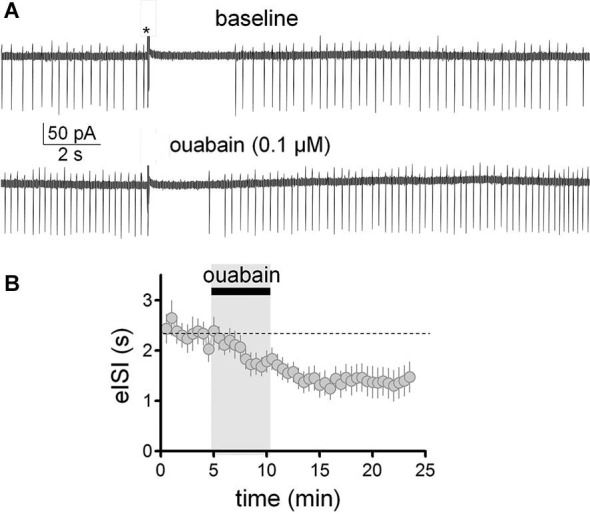
**Partial inhibition of the Na^+^/K^+^ pump with ouabain mimicked the effect of ethanol on the firing pause of Golgi cells. (A)** Sample traces corresponding to a loose-patch cell-attached recording from a Golgi cell. The baseline trace illustrates the pause in spontaneous firing of the Golgi cell induced by granule cell axon stimulation with a train of five stimuli (50 µs duration each at 100 Hz; marked with an asterisk). The lower trace shows that a submaximal concentration of the inhibitor of the Na^+^/K^+^ pump, ouabain (0.1 µM), increased basal firing of the Golgi cell and reduced the duration of the pause. **(B)** Pooled data from 13 Golgi cells illustrating the time course of the effect of ouabain on the pause duration (evoked interspike interval; eISI).

We have previously shown that slight inhibition of the Na^+^/K^+^ pump with a low concentration of ouabain (0.1 µM), mimics the effect of 40 mM ethanol on the membrane potential and spontaneous action potential firing of GoCs (Botta et al., 2010). Therefore, we tested whether this agent could also mimic ethanol’s effect on the firing pause (Figure 6). These experiments were performed in the loose-seal cell-attached configuration. In agreement with our previous findings (Botta et al., 2010), application of ouabain (0.1 µM) increased the firing frequency by 0.8 ± 0.2 Hz (Figure 6A; one sample *t*-test vs. 0, *t* = 3.97, *df* = 12, *p* = 0.002; *n* = 13). In addition, ouabain significantly decreased the firing pause (Figure 6B; baseline eISI = 2.38 ± 0.22 s; ouabain eISI = 1.37 ± 0.28 s; paired student *t*-test, *t* = 3.45, *df* = 12, *p* = 0.005; *n* = 13). In agreement with our previous report (Botta et al., 2010), the effect of ouabain was irreversible, as expected from the high affinity of this agent for the Na^+^/K^+^ pump.

## Discussion

Studies from multiple laboratories indicate that GoCs are important targets of ethanol. Acute ethanol exposure depolarizes the membrane potential and increases spontaneous action potential firing in these cells (Freund et al., 1993; Carta et al., 2004; Botta et al., 2010, Botta et al., 2012; Huang et al., 2012). Results of electrophysiological and/or computer modeling experiments suggest that the mechanism responsible for this effect involves, at least in part, ethanol-induced inhibition of the Na^+/^K^+^ pump and perhaps also a quinidine-sensitive K^+^ channel (Botta et al., 2010, Botta et al., 2012). A recent study suggests that ethanol-induced inhibition of neuronal nitric oxide synthase activity in GoCs also contributes to the mechanism of action of ethanol (Kaplan et al., 2013). The ethanol-induced increase of GoC excitability ultimately results in an increase in phasic and tonic GABA_A_ receptor-mediated currents in granule cells (Carta et al., 2004; Botta et al., 2010, Botta et al., 2012; Diaz et al., 2013; Kaplan et al., 2013). A recent study suggests that ethanol acts via an alternative mechanism where potentiation of *δ* subunit-containing extrasynaptic GABA_A_ receptors in granule cells leads to an increase in glutamatergic synaptic transmission in GoCs, indirectly leading to an increase in spontaneous action potential firing of these cells (Santhakumar et al., 2013). The increase in both phasic and tonic GABA_A_ receptor-mediated inhibition in granule cells is thought to be one of the mechanisms explaining the findings of *in vivo* electrophysiological recordings demonstrating a reduction in responsiveness of granule cells to mossy fiber inputs during acute exposure to ethanol (Huang and Huang, 2007).

Here, we report a novel effect of ethanol that could further limit the responsiveness of granule cells to incoming information transmitted by mossy fibers. We found that ethanol suppresses the pause in GoC firing triggered by high frequency stimulation of granule cell axons. This pause transiently suppresses spontaneous GoC firing, temporarily decreasing synaptic transmission at GoC-granule cell synapses (and perhaps also a portion of currents mediated by extrasynaptic GABA_A_ receptors), allowing strong mossy fiber inputs to more effectively excite granule cells, which then relay the signal to Purkinje cells via the parallel fibers and ascending axons (Figure 1A). Granule cells are thought to function as a high signal-to noise-filter because these cells display low levels of spontaneous firing activity as a consequence of the strong GABA_A_ receptor-mediated phasic and tonic inhibitory inputs (Chadderton et al., 2004). Incoming mossy fiber burst activity has been shown to effectively excite granule cells and this may facilitate the transmission of subsequent stimuli via transient inhibition of spontaneous GoC firing (Chadderton et al., 2004). Ultimately, the ethanol-induced reduction in the duration of the GoC pause is expected to result in increased filtering of mossy fiber inputs at the gateway of information into the cerebellar cortex. As mentioned above,* in vivo* studies have shown that GoC firing decreases in response to sensory inputs from the periphery and it would be interesting to determine whether this effect is reduced by acute ethanol exposure (Holtzman et al., 2006).

Given that mGluR2 has been shown to play a central role in the mechanism by which parallel fiber input induces the pause in GoC firing (Watanabe and Nakanishi, 2003), we evaluated the potential involvement of this receptor in the action of ethanol. Using juvenile (P14–P18) transgenic mice expressing the fusion protein of human interleukin-2 receptor subunit and GFP in GoCs, Watanabe and Nakanishi (2003) provided evidence suggesting that mGluR2 are primary mediators of the pause in GoC firing. In P23-P26 rats, we found that the mGluR2 antagonist, LY341495, only partially reduced the GoC firing pause. This is unlikely due to incomplete blockade of the receptor by LY341495 because we used a saturating concentration (1 µM); the IC50 of this agent for mGluR2 was reported to be near 20 nM (Kingston et al., 1998). Moreover, Watanabe and Nakanishi (2003) reported that a 0.5 µM concentration of this agent completely blocks mGluR2-mediated currents in GoCs of these mice and we also found near complete block in rat slices. In agreement with these findings, we found that 0.5 µM LY341495 completely blocked the DCG-IV-evoked currents under our experimental conditions. Therefore, it is possible that the mechanism of the GoC pause induction differs between Sprague-Dawley rats and the transgenic mice used by Watanabe and Nakanishi (2003), involving other players besides the mGluR2 in GoCs from Sprague-Dawley rats.

Two pieces of evidence suggest that changes in mGluR2 function do not play a role in the mechanism of action of ethanol. First, ethanol reduced the GoC pause to a similar extent in the absence and presence of LY341495. Second, ethanol did not have a significant effect on DCG-IV-evoked currents in GoCs. Based on studies indicating that ethanol potentiates GIRK channel function (Kobayashi et al., 1999; Lewohl et al., 1999; Aryal et al., 2009), it would have been expected for it to enhance DCG-IV-evoked currents. However, this would have resulted in lengthening of the pause. Although we observed potentiation of DCG-IV-evoked currents in some GoCs, these were not affected in most recordings. The reasons for the lack of sensitivity of DCG-IV-evoked currents to ethanol in GoCs are unknown but could involve differences in the characteristics of GIRK channels expressed in GoCs vs. those expressed in other neuronal populations (e.g., posttranslational modifications and association with other proteins). Taken together, these findings suggest that ethanol reduces the duration of the GoC pause by a different mechanism that does not involve modulation of mGluR2 or mGluR2-coupled GIRK channels. However, it should be emphasized that the effect of ethanol on the function of mGluR2 and GIRK channels in GoCs should be further characterized. For instance, it is possible that higher concentrations of ethanol more consistently potentiate mGluR2-evoked GIRK currents. It will also be interesting to assess the effect of ethanol on mGluR2-evoked GIRK currents elicited by release of glutamate from granule cell axons.

A novel finding of our study is that the GoC pause can be regulated by changes in the membrane potential and/or action potential firing frequency. We found that injection of depolarizing current caused a decrease in pause duration and injection of hyperpolarizing current increased the pause duration. This effect could be caused, in part, by changes in the function of receptors and/or channels involved in pause generation triggered by changes in the membrane potential. Another possibility is that increases in spontaneous action potential firing results in changes in [Ca^2+^]_*i*_ and other intracellular messengers, ultimately resulting in alterations in the receptors and/or channels involved in the generation of the pause. Clearly, the mechanisms responsible for this phenomenon require further investigation. Nevertheless, it is possible that both the ethanol-induced depolarization of the membrane potential and the action potential firing increase contribute to the mechanism by which ethanol reduces the firing pause. In support of this possibility is our finding that a submaximal concentration of ouabain, which we previously demonstrated to cause a similar change to that of ethanol in the membrane potential and firing frequency of GoCs (Botta et al., 2010), mimicked the effect of ethanol on the GoC pause. This finding suggests that ethanol acts, at least in part, by inhibiting the Na^+^/K^+^ pump in GoCs. It should be emphasized that we previously demonstrated that acute ethanol exposure increases GoC firing in a dose-dependent manner, producing significant effects at concentrations of 20, 40, and 80 mM (Botta et al., 2010). Therefore, other concentrations of ethanol (i.e., 20 and 80 mM) are expected to inhibit the GoC pause in addition to the 40 mM concentration tested in this study.

In conclusion, we have identified a novel effect of ethanol on GoC physiology that involves a reduction in a feedback mechanism that normally results in a transient decrease of GABA_A_ receptor-mediated inhibition of granule cells. This effect may contribute to the decrease in granule cell responsiveness to mossy fiber input that is observed during acute ethanol exposure (Huang and Huang, 2007). Together with other effects of acute ethanol exposure on several cellular components of cerebellar cortical circuits (Botta et al., 2007; Valenzuela et al., 2010), this effect may contribute to produce the motor and cognitive alterations that are associated with acute ethanol intoxication. It was recently shown that the mGluR2-mediated hyperpolarization of the membrane potential can, under some conditions, trigger a rebound long-term increase in the frequency of spontaneous action potential firing in GoCs (Hull et al., 2013). Future studies should examine whether this firing rate plasticity mechanism is also impaired by ethanol.

## Author contributions

Paolo Botta and C. Fernando Valenzuela designed experiments, analyzed data and prepared the manuscript. Paolo Botta and Aya Zucca conducted the experiments.

## Conflict of interest statement

The authors declare that the research was conducted in the absence of any commercial or financial relationships that could be construed as a potential conflict of interest.
